# Effect of Preheat Temperature and Welding Sequence on the Temperature Distribution and Residual Stress in the Weld Overlay Repair of Hydroturbine Runner

**DOI:** 10.3390/ma15144867

**Published:** 2022-07-13

**Authors:** Jimiao He, Min Wei, Lixin Zhang, Changrong Ren, Jin Wang, Yuqi Wang, Wenkai Qi

**Affiliations:** 1 College of Mechanical and Electrical Engineering, Shihezi University, Shihezi 832003, China; 20202109073@stu.shzu.edu.cn (J.H.); zhlx2001329@163.com (L.Z.); 20202109069@stu.shzu.edu.cn (C.R.); wangjin1@stu.shzu.edu.cn (J.W.); 20202109023@stu.shzu.edu.cn (Y.W.); 2 Hongshanzui Power Plant, Xinjiang Tianfu Energy Co., Ltd., Shihezi 832003, China; qwk17860772875@163.com

**Keywords:** hydroturbine runner, preheat temperature, welding sequence, welding residual stress

## Abstract

The hydroturbine runner is the core of the whole hydroelectric generating unit, which is employed to transform water energy into mechanical energy. In the process of service, the runner frequently suffers from abrasion due to erosion and cavitation. Defects are usually repaired by welding. To acquire suitable weld cladding repair process parameters, a combination of experimental and numerical simulation was applied to investigate the temperature and weld residual stress distribution in the repair zone under the different welding repair approaches. The results illustrate that the temperature and welding residual stress distribution of the blade and the shroud are out of symmetry, the temperature conduction rate is faster on the blade side, and the high-stress zone is predominantly concentrated in the weld and its adjacent area. When the preheating temperature is up to 150 °C, the peak value of welding residual stress reaches a minimum of 796.29 MPa. The welding sequence can adjust the distribution trend of welding residual stresses. The welding sequence of three-stage welding can effectively reduce the welding residual stresses near the shroud at the water outlet side of the blade. The results of the study will provide theoretical guidance for the welding repair of hydraulic turbine runners.

## 1. Introduction

The hydroturbine runner is the core of the entire hydroelectric generating set, which includes [1]: the upper crown, shroud, and blades. The blade is the most important component of the runner, and its quality directly impacts the working efficiency and safe and stable operation of the hydraulic turbine. The maximum principal stress and the maximum bending stress of the hydraulic turbine runner are mainly concentrated in the blade and the shroud or the upper crown connection, which are prone to abrasion after erosion by high-speed sand-laden water and cavitation [2,3,4]. In particular, the blade and the shroud connection near the water outlet edge, where the location is thinner, the hydraulic turbine runner in the service process is more prone to abrasion. It is necessary to repair the abraded area in time; otherwise, it may lead to catastrophic accidents and cause huge economic losses.

At present, welding overlay repair is mainly used for the abraded area of the hydroturbine runner [5,6]. Due to the advantages of flexible operation, strong adaptability, and a wide range of applications, welding overlay has already become an essential means of repairing local defects in engineering institutes. Welding overlay is a complex process involving the intersection of heat transfer, material metallurgy, mechanics, and other disciplines. The rapid local heating and cooling during the welding process results in uneven thermal expansion and contraction of the workpiece, which inevitably leads to residual stress and deformation of the workpiece so that the repair quality of the welded structure decreases [7,8,9,10]. 0Cr13Ni5Mo is a common material for hydraulic turbine runners, which is an ultra-low carbon martensitic stainless steel. In order to avoid the tendency of cracking and to improve the toughness during welding repair, it is necessary to choose welding materials with good weldability and wettability. In comprehensive consideration, this paper selects the 0Cr13Ni5MoRe stainless steel electrode of the same material as the base metal. The hydroturbine blade is a three-dimensional twisted surface body, and the residual stress distribution generated during the welding process is very complex. Therefore, the key point to be solved is how to properly regulate residual stress and deformation of the welded junction between the blade and the shroud to ensure the hydraulic turbine’s safe and stable operation. Many researchers have previously investigated residual stresses and deformations in thick plate butt welds [11,12,13,14], longitudinal welds [15,16,17], and dissimilar steels [18,19,20]. For example, the welding sequence had a substantial effect on the plate deformation and longitudinal stress distribution induced by welding [21], according to Chen et al. Fallahi et al. [22] discovered that preheating and proper welding sequences were effective in reducing residual stresses during welding. Shao et al. [23] found that the preheating temperature and welding speed had the greatest impact on the welding stress and deformation of aluminum alloy T-joints, followed by the preheating area. Peric et al. [24] studied the effects of different preheating temperatures and inter-pass times on the residual stress and deformation of the welded structure. The results showed that the preheating temperature applied before the start of welding could significantly reduce the post-weld deformation of the welded structure. However, as the inter-pass time increased, the deformation of the plate increased. Mai et al. [25] found that adjusting the welding sequence can change the distribution trend of residual stress in saddle-shaped welds. Yang et al. [26] discovered that by adjusting the welding sequence, the welding residual stress of the SA738 thick plate can be effectively reduced. Lee et al. [27] investigated the influence of preheating and joint geometry on the distribution of residual stresses near the weld toe of plate-to-plate T-joints and Y-joints using the ASTM drilling method and found that preheating is an effective way to reduce weld residual stresses. Charkhi et al. [28] investigated the use of the preheating process in steel pipe repair welding and discovered that increasing the preheating temperature of the repaired welded pipe reduced longitudinal residual stresses on the inner and outer surfaces of carbon and stainless-steel pipes by 35–50%, respectively, but had no effect on compressive residual stresses on the outer surfaces. Zhang et al. [29] adopted a fiber laser to weld a thin-walled nanostructured molybdenum alloy tube and found that the maximum weld residual tensile stress decreased monotonically with increasing preheat temperature. Xu et al. [30] discovered that as the preheating temperature increased, the peak circumferential stresses decreased. However, the heat input had almost no influence on the residual stresses in the tube-to-plate welds. Lee et al. [31] discussed the welding temperature field and residual stress distribution of Q345/SUS304 flat plate butt joints based on the finite element model and test results. Scholars mainly do research on relatively regular substrates such as flat plates and pipes, but less research on actual welded structures. The hydraulic turbine blade is a three-dimensional twisted curved body, and the structure is very complex. In the welding repair process, there is a complex stress field. As a result, the research and analysis of the temperature distribution during the welding repair process and the transient temperature field, stress field, and residual stress after welding are of great significance to optimize the welding process and improve the quality of the welding repair in this paper.

In this paper, welding experiments were performed on the connection between the hydroturbine blade and the shroud. In addition, the welding numerical simulation and analysis of the connection between the turbine blade and the shroud is carried out using the thermo-mechanical coupling method, and the acquired results have been compared to the experimental measurement results to verify the welding numerical simulation method’s reliability. The influence of alternative welding sequences and preheating temperatures on the temperature changes and residual stress distribution trend in the weld at different places of the blade and lower ring joint was compared and assessed using the welding numerical simulation results of the finite element model. Finally, the optimum welding repair process parameters for the failure area at the blade and lower ring joints were determined, and the results of the study provide theoretical guidance for the welding repair process of the hydraulic turbine runner.

## 2. Experimental Procedure

**Figure 1:**
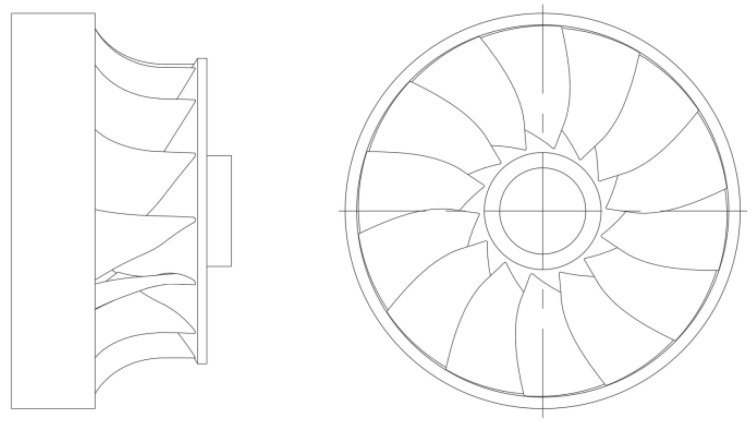
CAD schematic of the hydraulic turbine runner structure.

**Figure 2:**
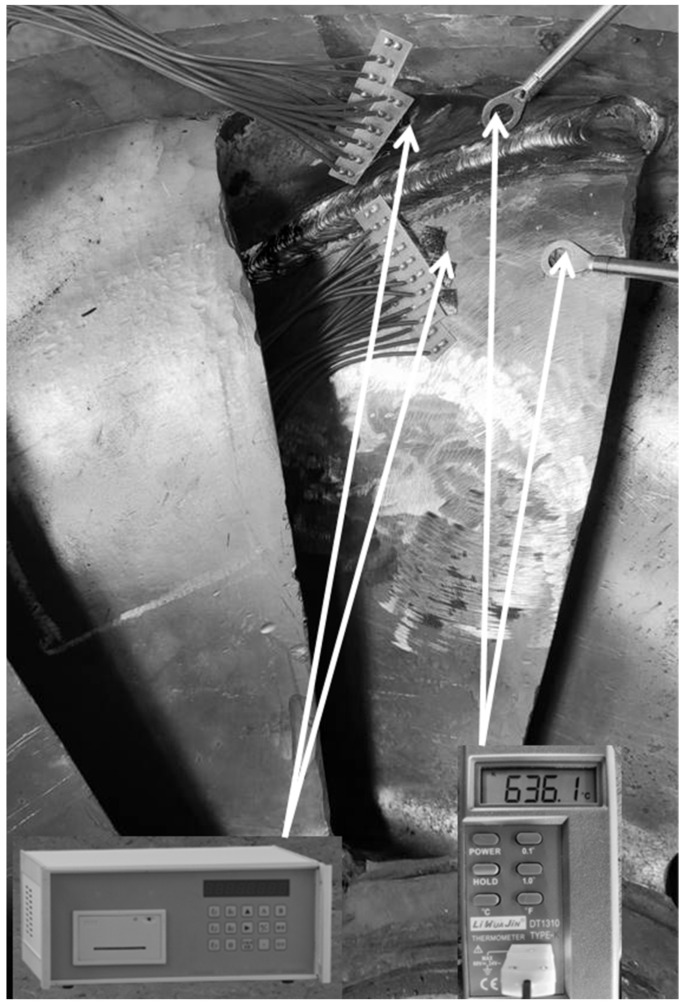
Experimental locations with hole drilling method.

Welding experiments were carried out using shielded metal arc welding (SMAW) on the connection between the hydroturbine blade and the shroud. Figure 1 is a two-dimensional schematic diagram of the runner structure. The entire welding area is divided equally into three sections along the welding direction for welding repair. First, complete the welding of the welding section A_1_ at the water outlet, starting the arc 5mm from the water outlet of the blade, and then complete the water inlet welding section B_2_, beginning the arc at 5 mm from the water inlet. Finally, the middle part of C_3_ is welded, starting the arc on the side near the water inlet side of the blade and closing the arc on the side near the water outlet side of the blade. The welding sequence adopted three-stage welding. The material of the runner is 0Cr13Ni5Mo, which is martensitic stainless steel. The filler metal is 0Cr13Ni5MoRe stainless steel welding rod with Ø3.2 mm. The material composition of the base material and the filling metals are listed in Table 1. Choose an AoTai welding power supply. The maximum output current is 450 A. Filler metal is sensitive to impurities and prone to welding defects. Therefore, the substrate should be cleaned before the welding repair. To begin, clear the oxide coating and debris from the substrate’s surface using a wire brush, then scrub it with acetone and dry it; the surface of the electrode is polished with fine sandpaper to remove impurities from the electrode surface. Finally, the welding repair experiment is carried out according to the predetermined experimental scheme. Figure 2 is a schematic diagram of the hydraulic turbine runner welding experiment. In the welding process, the welding current is 210 A, the welding voltage is 22 V, the welding speed is 5 mm/s, and the welding polarity is DC reverse. To avoid cracking and heat-affected zone embrittlement of martensitic stainless steel during welding, the base material is uniformly heated by means of oxy-acetylene flame gun heating. The preheating range is 50 mm on both sides of the connection between the blade and the shroud. The preheating temperature is 150 °C and the temperature is measured by a thermocouple. Due to the lower preheating temperature, the microstructure does not change during the process. In addition, the weld residual stresses in the welded structure are measured using the blind hole method (borehole strain method) after the welding is completed.

## 3. Numerical Simulation

General finite element simulation software Simufact Welding 6.0.0 was employed. A 3D finite element model consistent with the actual weld sample is established based on thermal-elastic-plastic theory to acquire the temperature and residual stress evolution law of the weld sample during the welding process. In order to obtain accurate simulation results, the thermal-mechanical coupling method was adopted. The filler process of the welding seam is processed by using the life-and-death element technology. The welding filler is gradually filled up to the welding seam. Therefore, in the numerical simulation process, the element in the unwelded area is inactive, and as the welding process progresses, the element in the area where the welding heat source is placed is activated, improving the accuracy of the simulation results.

### 3.1. 3D Modeling and Meshing

**Figure 3:**
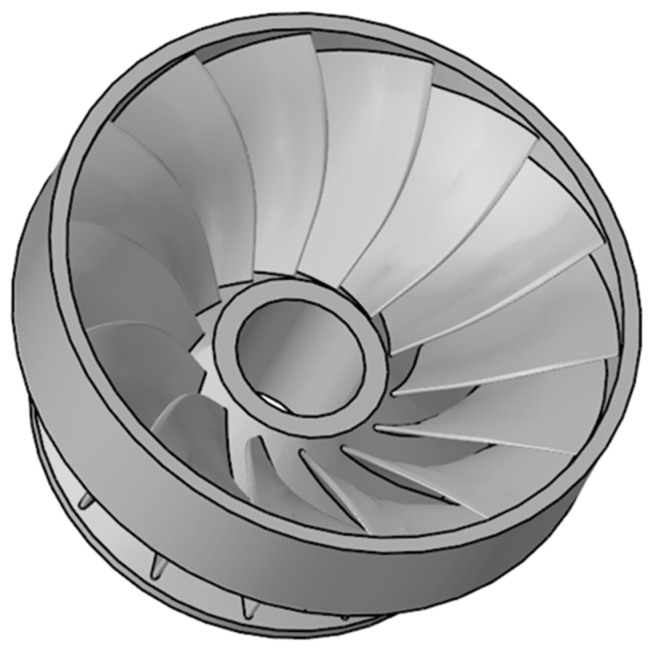
3D models of runner.

**Figure 4:**
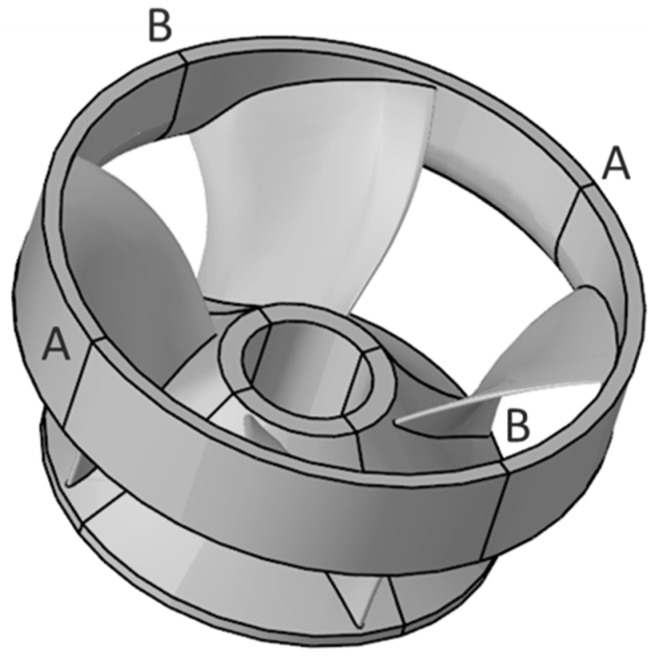
3D models of simplified runner.

**Figure 5:**
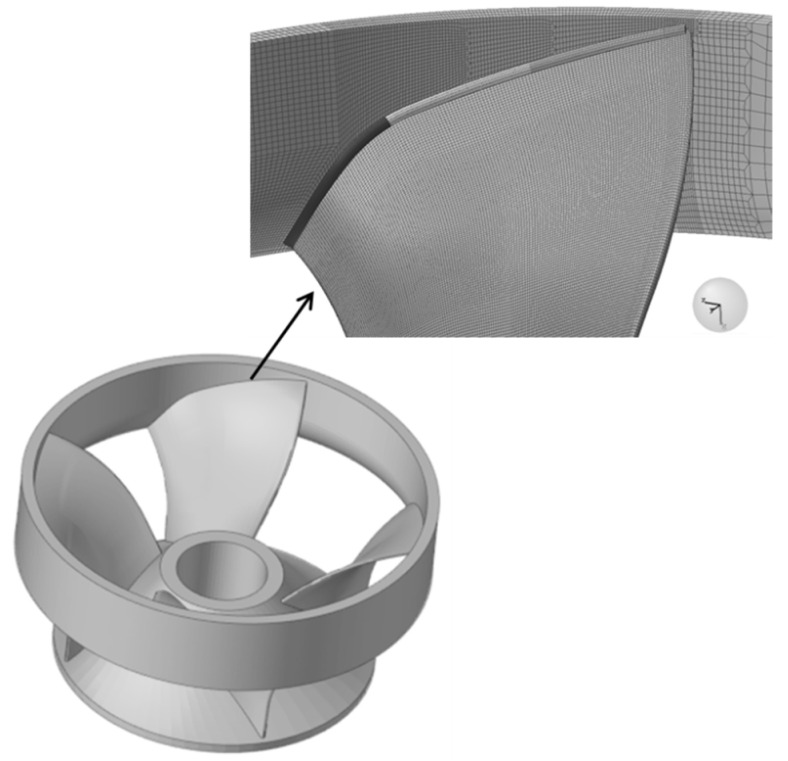
Runner 3D Finite Element Mesh.

In view of the fact that the modeling function of Simufact Welding 6.0.0 itself is not very strong. Therefore, this paper uses Solidworks 3D solid modeling software to establish the same 3D model of the runner as the original welded parts, as illustrated in Figure 3. After the model has been built, save it to SLDPRT format and import it to the Hypermesh component in Hyperworks software for surface meshing and body meshing. It is very difficult to directly simulate the runner numerically as it features a large structure and a large number of blades, so the model needs to be simplified. The blades are evenly distributed. Therefore, four blades are taken, one of which is used as the object of study, and the other three are used as supports to ensure the overall stiffness of the model. The simplified model of the runner is shown in Figure 4. For the purpose of improving the efficiency of meshing, the 3D model is cut along the A-A and B-B sections, and a quarter of the 3D model is taken for meshing. The blade, the shroud, and the upper crown are divided into surface meshes and, in turn, the area close to the weld is divided into fine mesh and the area far from the weld is divided into sparse mesh. Finally, the solidmap command is used to mesh the body, and hexahedral elements are selected for the body mesh type. The mesh division of the runner finite element model is depicted in Figure 5.

### 3.2. Heat Source Model

The heat source model is the basis of the finite element analysis of welding, and its selection directly affects the accuracy of the simulation results. This study adopts the double ellipsoid heat source model proposed by Goldak et al. [32], which can better restore the real welding pool and is closer to the real welding situation. Adjust the heat source parameters according to the actual situation. The adjusted heat source parameters *a_f_*, *a_r_*, *b*, *d* are 2.8, 7.6, 3.5, and 4.85 mm, respectively. The heat flow density distribution of the double ellipsoid heat source model can be described by the following two mathematical Equations.

The distribution of heat flux density in the front quadrant of the double ellipsoid is *q_f_* (*x*, *y*, *z*, *t*) (1):

(1) $$q_{f}\left(x,y,z,t\right)=\frac{6\sqrt{3}f_{f}Q}{a_{f}bc\pi \sqrt{\pi }}\exp\left(-\frac{3x^{2}}{a_{f}{}^{2}}-\frac{3y^{2}}{b^{2}}-\frac{3{\left[z+v\left(\tau -t\right)\right]}^{2}}{c^{2}}\right)$$

The distribution of heat flux density in the rear quadrant of the double ellipsoid is *q_r_* (*x*, *y*, *z*, *t*) (2):

(2) $$q_{r}\left(x,y,z,t\right)=\frac{6\sqrt{3}f_{r}Q}{a_{r}bc\pi \sqrt{\pi }}\exp\left(-\frac{3x^{2}}{a_{r}{}^{2}}-\frac{3y^{2}}{b^{2}}-\frac{3{\left[z+v\left(\tau -t\right)\right]}^{2}}{c^{2}}\right)$$

where *f_f_* and *f_r_* are the shares of heat input in the two-ellipsoid front and back, and *f_f_* + *f_r_* = 2, *Q* = ηUI, *Q* is the heat source power, U is the welding voltage, I is the welding current, *v* is the welding speed, and η is the welding heat efficiency. Heat loss is unavoidable in the welding process, so η = 0.75; where *a_f_* and *a_r_* denote the front and rear semi-ellipse lengths of the double ellipsoidal heat source model, respectively; where *b* and *c* represent the width and depth of the heat source model, respectively.

### 3.3. Thermal Analysis

The thermal-physical properties of the material change with the continuous change of temperature during the welding process. Therefore, the finite element of welding temperature field analysis is typically a non-linear transient heat transfer problem. In accordance with Fourier’s heat transfer theory and the law of conservation of energy, the heat conduction governing equation of the transient temperature field of the welding process can be expressed by the following Equation (3):

(3) $$c\rho \frac{\partial T}{\partial t}=\frac{\partial }{\partial X}\left(\lambda \frac{\partial T}{\partial X}\right)+\frac{\partial }{\partial Y}\left(\lambda \frac{\partial T}{\partial Y}\right)+\frac{\partial }{\partial Z}\left(\lambda \frac{\partial T}{\partial Z}\right)+Q\left(X,Y,Z,t\right)$$

where *c* is the specific heat capacity (J·kg^−1^·°C); *ρ* is the density of the material (kg/m^3^); *λ* is the thermal conductivity (W·m^−1^·°C); *T* represents the temperature field distribution function (°C); *Q* (*x*, *y*, *z*, *t*) represents the heat generated or consumed per unit time (W); and *t* represents the heat transfer time (s); Both *c* and *λ* of these parameters change with temperature.

Heat losses due to convection and radiation are considered for all workpiece surfaces and thermal boundary conditions. Thermal convection is the process of transferring internal energy from a higher to a lower temperature part of the workpiece surface when it comes into contact with a fluid. During the welding process, the heat convection between the liquid in the molten pool and the surrounding solid can be described by Newton’s cooling Equation (4):

(4) $$Q_{C}=\left(T_{S}-T_{B}\right)h$$

where *h* is the convective heat transfer coefficient, *T_S_* is the surface temperature of an object, and *T_B_* is the temperature of the surrounding fluid.

Thermal radiation is the process in which objects emit energy directly into other objects without relying on a medium. Radiation loss mainly occurs in the fusion zone and near the high-temperature area. The intensity value of thermal radiation is related to the temperature of the object itself. The relationship for thermal radiation can be described by the following mathematical Equation (5):

(5) $$q=24.1\times 10^{-4}\varepsilon T^{1.61}$$

where *ε* is the radiative heat transfer coefficient, *ε* = 0.82; *T* is the absolute temperature.

### 3.4. Mechanical Analysis

In the welding process, the welding heat input will inevitably result in greater stress and deformation of the welded parts. The total strain of the material includes elastic strain, thermal strain, plastic strain, phase transformation plastic strain, and creep strain [33]. Due to the short high-temperature residence time during the welding process, the creep strain can be neglected. The total strain of the material can be described by the following governing Equation (6):

(6) $$\varepsilon^{total}=\varepsilon^{e}+\varepsilon^{p}+\varepsilon^{t}+\varepsilon^{Tr}$$

where *ε^e^* is the elastic strain increment, *ε^p^* is the plastic strain increment, *ε^T^* is the thermal strain increment, *ε^Tr^* is the strain increment of transformation-induced plasticity (TRIP). During numerical simulations, thermal strain is treated as a strain caused by the coefficient of thermal expansion and phase change. The elastic strain is modeled using isotropy and obeys Hooke’s rule, and the yielding behavior of the material follows the Mises norm.

### 3.5. Determination of Material Parameters and Boundary Conditions

**Figure 6:**
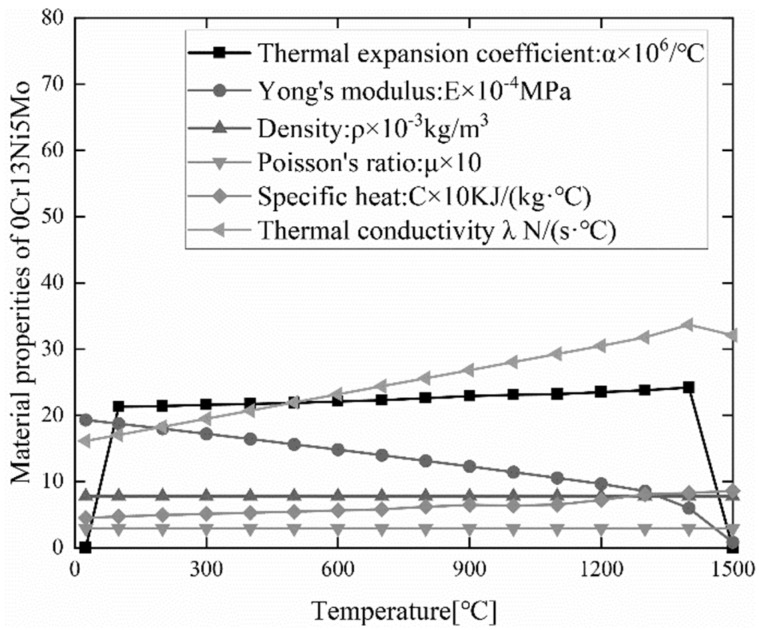
Thermal and mechanical properties of 0Cr13Ni5Mo.

In the welding finite element simulation process, the thermal-physical properties of the material parameters have a great impact on the simulation results. In this paper, the temperature-dependent thermal and mechanical properties of 0Cr13Ni5Mo are taken into account. The temperature-dependent specific heat capacity, density, Poisson’s ratio, coefficient of thermal expansion, and other material properties of 0Cr13Ni5Mo were obtained by defining the temperature range using the professional material calculation software JMatPro. The filler metal material 0Cr13Ni5MoRe has a small difference in performance with the base material 0Cr13Ni5Mo. Therefore, the thermal-mechanical properties of the filler metal remain consistent with the base material during the numerical simulation of welding. The thermal-mechanical property parameters of 0Cr13Ni5Mo steel are shown in Figure 6. The model is placed on a rigid support platform depending on gravity. In addition, no constraints are imposed throughout the welding process, matching the welding experiment boundary conditions.

### 3.6. Determination of Welding Case

**Figure 7:**
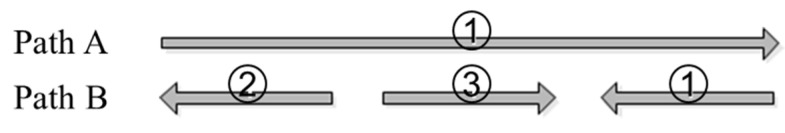
Two welding sequences.

Considering that the water outlet edge of the hydroturbine blade is thin, there is a complex stress distribution, and it is easy to deform after welding. In order to ensure the dimensional accuracy of the turbine blades after welding, two welding sequences are proposed based on the welding sequence under traditional process conditions and combined with the operability in the production process, as shown in Figure 7. Welding path A is continuous welding. The arc starts at 5 mm from the water inlet edge of the blade, and the arc ends at 5 mm from the water outlet edge of the blade. Welding path B is three-stage welding, and the arc starting and ending positions are the same as in the welding experiment. The arrows in the diagram represent the welding direction of the torch, and the numbers represent the welding sequence. In addition, in order to investigate the influence of the preheating temperature on the temperature and stress fields in the repair area, three groups of preheating temperatures were set up for numerical simulation on the basis of the above two welding sequences. The welding case is shown in Table 2, and the welding parameters are consistent with the data used in the welding experiment.

### 3.7. Thermal Cycle, Residual Stress Measuring Point Layout

**Figure 8:**
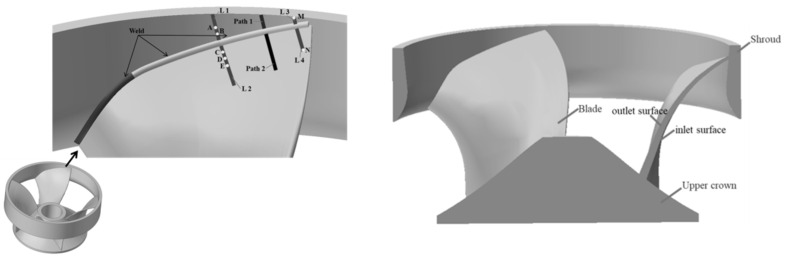
Thermal cycle, stress measurement point and path layout.

Figure 8 shows the thermal cycle, residual stress measurement points and path layout. Path 1 and Path 2 are located at the midpoint of the water outlet welding section A_1_ is perpendicular to the centerline of the water outlet welding section A_1_. L1 and L2 are located at the arc end of the C_3_ welding section in the middle part, and the characteristic points B and A are, respectively, 5 and 10 mm away from the center line of the weld; feature points C, D, and E are 5, 10, and 15 mm away from the centerline of the weld, respectively; L1 and L2 are 30.5 mm away from Path 1 and Path 2, the feature point M is 6.5 mm from the center line of the weld, and the feature point N is 15.5 mm from the center line of the weld. The characteristic points A, B, C, D, and E are used to measure the welding residual stress, and the characteristic points M and N are used to measure the welding thermal cycle.

## 4. Experimental Validation of the Numerical Simulation Mode

In order to demonstrate the validity of the welding finite element model, the welding finite element model needs experimental validation. In this paper, welding experiments were performed on Case 6 in Table 2, by using K-type thermocouples to measure the transient temperatures at different locations near the water outlet weld section A_1_. The thermocouple is positioned on the blade and lower ring surface as shown in Figure 8. They are located on the blade at 15.5 mm from the center line of the weld and on the shroud at 6.5 mm from the center line of the weld, respectively. Post-weld residual stresses are measured at specified locations using the blind hole method (borehole strain method). The location of the residual stress measurement is shown in Figure 8. The welding finite element simulation results are compared with experimental measurements to verify the validity of the welding finite element model.

### 4.1. Thermal Cycling Curve Verification

**Figure 9:**
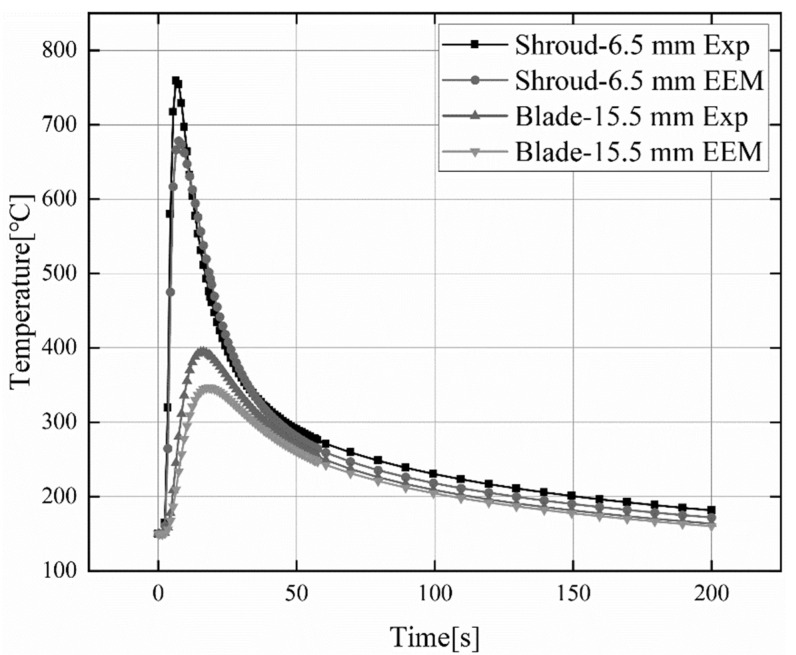
Comparison of experimental and simulated values for transient temperature changes of shroud and blade surfaces.

Figure 9 indicates the simulation results and experimental results of transient temperature changes on the blade and lower ring surfaces. It can be observed that the simulated values at 6.5 mm and 15.5 mm from the center of the weld are slightly lower than the experimental values. The measured maximum temperature was 759.6 °C, and the simulated maximum temperature was 678.3 °C. When in the heating stage, the heating rate of the measured temperature value and the simulated temperature value is basically synchronized. However, during the cooling stage, the cooling rate of the simulated temperature value is slightly higher than the cooling rate of the measured temperature. In general, the measured temperature values are in good agreement with the simulated calculated values, and the change trends are generally consistent.

### 4.2. Welding Residual Stress Verification

**Figure 10:**
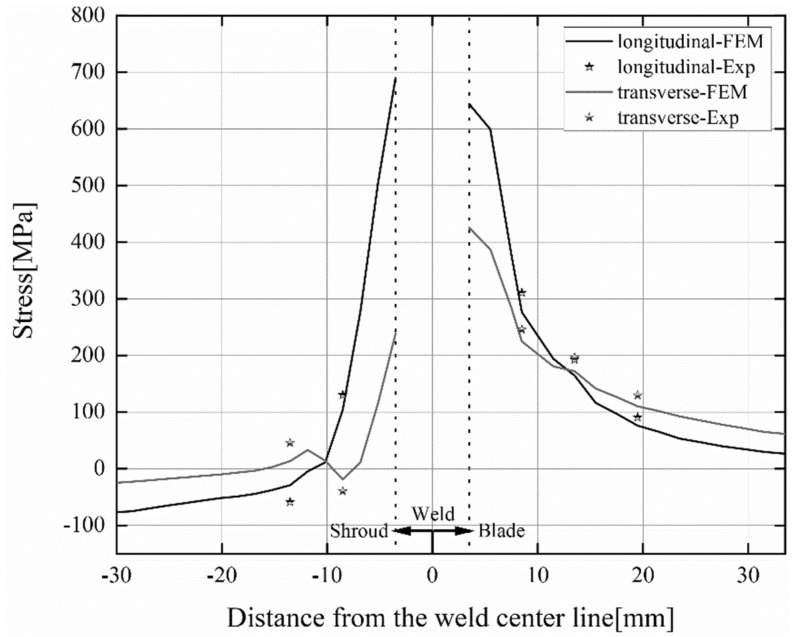
Experimental measurement results and numerical simulation results of post-weld residual stresses.

Three feature points were selected on the blade surface, two feature points were selected on the shroud surface, and a total of five feature points were measured for post-weld residual stress. The location of the characteristic points is shown in Figure 8. The numerical simulation and experimental results of longitudinal and transverse residual stresses are plotted in Figure 10. The left side of the origin of the coordinate system represents the residual stress on the shroud surface, and the right side represents the residual stress on the blade surface. It is difficult to reasonably measure the residual stress at the weld surface in the experiment, so the paper does not show the residual stress at the weld. It can be observed from Figure 10 that the longitudinal and transverse residual stress values calculated by the finite element model are in agreement with the change trend of the experimental measurement results, but there are certain deviations. The factors contributing to this deviation may be simulation and measurement. For numerical simulations, the difference in results is mainly due to the inaccuracy of the finite element model, the deviation of material property parameters, etc. For the measurement, the difference in the results is mainly the measurement error of the test measuring instrument, the hole depth error, the hole diameter error, and drilling eccentricity, etc. In addition, another reason may be the internal stress produced due to the erosion of the runner by the high-speed sandy water during service. In the welding repair process, the internal stresses produced during the service period will be indirectly combined with the stresses produced during the welding repair process, eventually having an impact on the trend of residual stress distribution. However, residual stresses produced during in-service are neglected in the FEM model. Overall, the numerical simulation results can better reflect the actual post-weld residual stress.

## 5. Results and Discussion

### 5.1. Analysis of Welding Temperature Field Results

**Figure 11:**
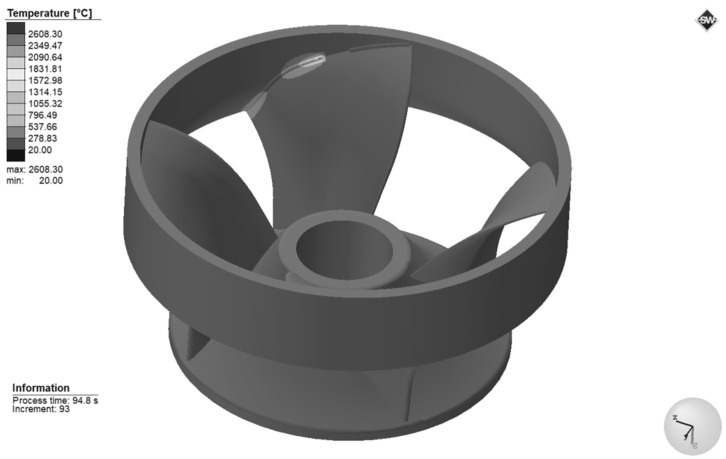
3D temperature profiles on the outer surface at 94.8 s.

The accuracy of the welding temperature field is the premise and basis for obtaining reasonable residual stress and deformation. Figure 11 represents the temperature field cloud diagram during the welding process at the moment of 94.8 s under Case6 conditions in Table 2. It can be observed that the filler metal is continuously activated as the heat source moves, which is in line with the actual welding process. The highest temperature is found in the weld’s center, which reaches 2608.3 °C, exceeding the material’s melting point. The weld seam and the surrounding area form an elliptical heat diffusion trajectory, spreading the heat outward continuously. The isotherm distribution in front of the heat source is dense, and the temperature gradient is large. However, the isotherm distribution in the rear of the heat source is sparse, and the temperature gradient is small.

**Figure 12:**
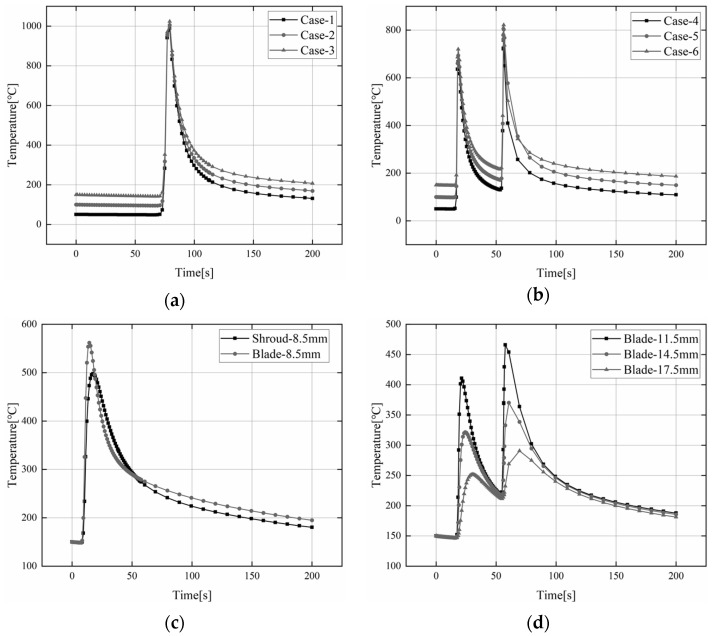
Transient temperature distribution. (**a**) Transient thermal cycling of Path A; (**b**) Transient thermal cycling of Path B; (**c**) Transient thermal cycle at the same distance from the center of the weld; (**d**) Transient thermal cycles at 11.5, 14.5 and 17.5 mm from the center of the weld.

Figure 12a–d depicts the transient temperature change trend near the connection between the blade and the shroud under six different welding processes (Case 1–Case 6). Due to the large temperature gradient changes in the initial welding period, only the transient temperature changes from 0–200 s are extracted. Figure 12a represents the transient temperature change trend at different preheating temperatures under Path A. Path A is continuous welding, and each thermal cycle curve has only one peak and one trough. Figure 12b represents the transient temperature change trend at different preheating temperatures under Path B. The characteristic point is located on L2 near the arc end of the welding section A_1_ at the water outlet and the welding section C_3_ in the middle part. Each thermal cycle curve has two peaks and two troughs, which indicate the characteristic point’s maximum and lowest temperatures, respectively. Figure 12c represents the transient temperature change trend of the blade side and shroud side at the same distance from the center of the weld. As can be seen, the peak temperature of the feature point on the blade side is higher than the temperature of the feature point on the lower ring side. That is due to the thinner blade and faster temperature conduction on the blade side. Figure 12d shows the transient thermal cycles at 11.5, 14.5 and 17.5 mm from the center of the weld. It can also be observed that the closer to the fusion zone, the higher the temperature peak. On the contrary, the farther away from the fusion area, the smaller the temperature peak, which is caused by the different heat inputs during the welding process.

### 5.2. Analysis of Welding Stress Field Results

**Figure 13:**
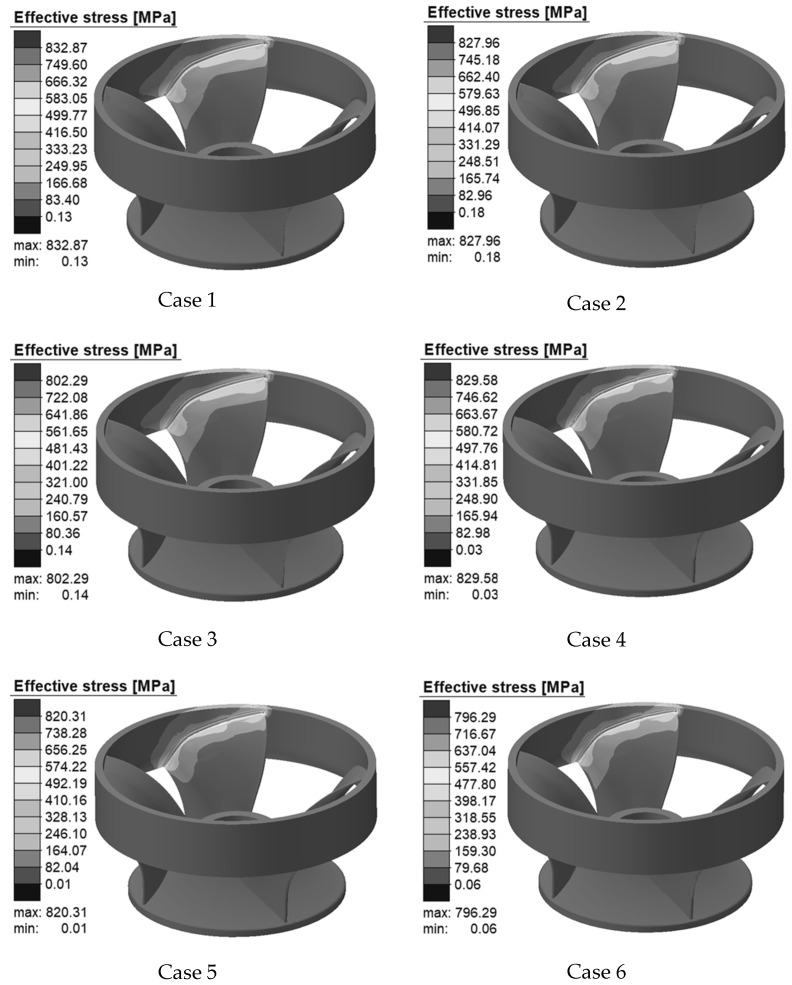
Von Mises stress distribution in each case.

Figure 13 represents the Von Mises stress distribution state of Case 1–Case 6 after welding cooling is completed. The overall distribution of welding stress is not uniform, and high-stress locations are mostly localized in the weld and nearby areas, with a substantial stress gradient. This is because the weld and adjacent areas are subject to large heat input, and the thermal stress generated by the thermal expansion of the metal is also large. On the contrary, the area away from the weld is subject to less heat input and generates less thermal stress. By comparing the peak equivalent stresses of Case 1–Case 6, it can be observed that with the increase in preheating temperature, the maximum value of stress decreases. Therefore, a reasonable preheating temperature can reduce the welding residual stress, which is of great significance for the welding repair of the hydroturbine runner.

**Figure 14:**
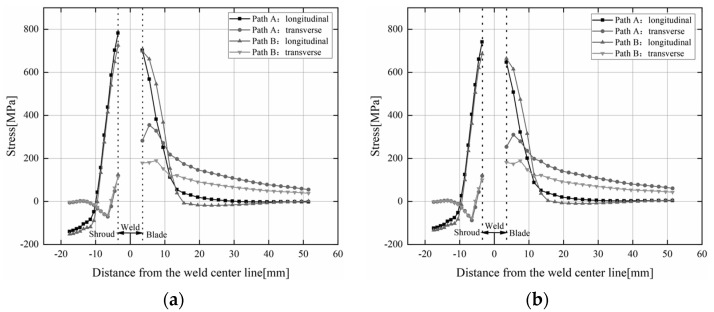
Transverse and longitudinal residual stresses along Path 1, Path 2. (**a**) Preheat—50 °C; (**b**) Preheat—150 °C.

In order to further study the effects of preheating temperature and welding sequence on residual stress at different positions in the repaired area, two welding sequences with a preheating temperature of 50 °C and 150 °C were selected for analysis. The longitudinal and transverse residual stress distributions of the two welding sequences are depicted in Figure 14a,b when the preheating temperature is 50 and 150 °C, respectively. It can be noticed that the trends of transverse and longitudinal residual stress distribution along Path 1 and Path 2 are basically the same under different preheating temperatures. The longitudinal residual stresses under the two welding sequences are not significantly different in value, while the transverse residual stresses in Path B are significantly smaller than those in Path A. There is a significant stress gradient near the weld or weld fusion line. The longitudinal and transverse residual stress peaks of Path A reached 782.2 and 355.3 MPa, respectively; the longitudinal and transverse residual stress peaks of Path B reached 725.3 and 190.1 MPa, respectively. When the preheating temperature was 150 °C, the longitudinal and transverse residual stress peaks of Path A reached 741.2 and 310.3 MPa, respectively; the longitudinal and transverse residual stress peaks of Path B reached 685.5 and 189.3 MPa, respectively. This is mainly because the heat input in the weld area is large, and the thermal stress generated by the thermal expansion of the metal is also large. At the shroud 17.5 mm from the centerline of the weld, the longitudinal residual stress of the two welding sequences is stable at −120 MPa, while the transverse residual stress tends to 0 MPa. At the blade 20 mm away from the centerline of the weld, the longitudinal and transverse residual stress changes of the two welding sequences gradually tend to be flat. In general, the welding sequence of Path B is better than that of Path A, and the residual stress peak decreases as the preheat temperature increases.

**Figure 15:**
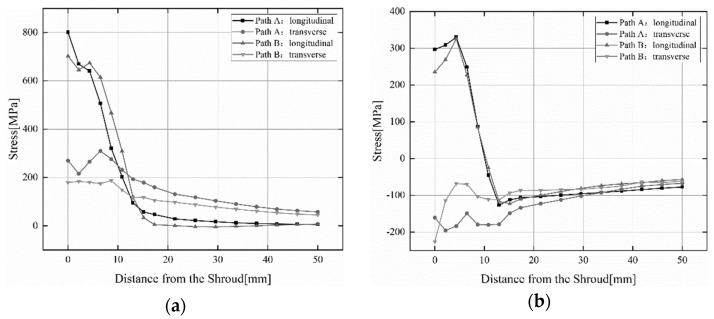
Residual stress distribution on the water outlet surface and water inlet surface of the blade with the same section. (**a**) water outlet surface; (**b**) water inlet surface.

In order to study the residual stress on the water outlet surface and water inlet surface of the blade with the same section, use the cutting tool in the post-processing window of the software to cut along the Y-axis at the midpoint of the center line of the A_1_ welding section at the water outlet, and measure the residual stress along the water outlet surface and water inlet surface of the blade, as shown in Figure 8. Figure 15 shows the longitudinal and transverse residual stress distributions on the water outlet surface and water inlet surface of the blade for two welding sequences when the preheating temperature is 150 °C. It can be observed that the blade out of the water surface residual stress is higher than the inlet surface, the transverse residual stress is lower than the longitudinal residual stress, and the longitudinal residual stress has a larger peak value. The directions of the longitudinal and transverse residual stress on the blade outlet surface are tensile stress, and the residual stress is generally monotonically decreasing in distribution. The direction of longitudinal residual stress on the blade water inlet surface at a distance of 10 mm from the shroud is tensile stress, and the direction of longitudinal residual stress beyond 10 mm from the shroud is compressive stress. The transverse residual stress direction of the blade inlet surface is completely opposite to the transverse residual stress direction of the blade outlet surface. The maximum value of transverse residual stress is about 330.1 MPa, which is about 53.2% of the yield strength of the blade material [34]. The distribution of longitudinal residual stresses in both Path A and Path B welding sequences remains basically the same. However, the transverse residual stress of Path B is significantly smaller than that of Path A, which is consistent with the conclusion drawn above.

**Figure 16:**
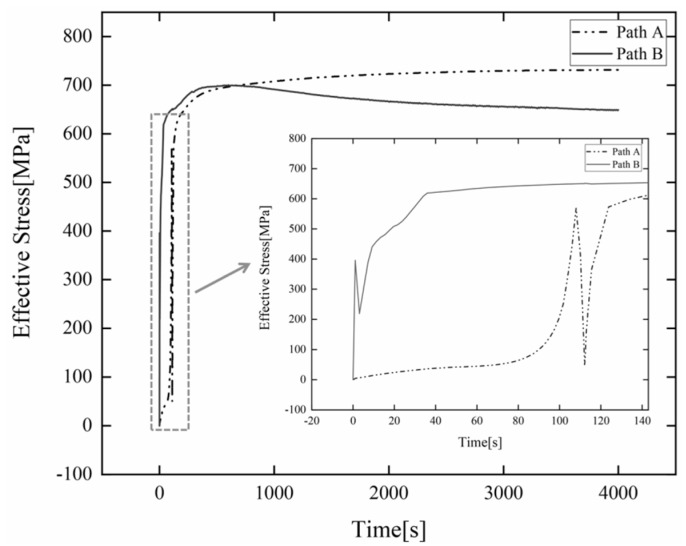
Variation of residual stress at the water outlet edge of the blade near the shroud.

The water outlet edge of the blade near the lower ring is the position where the hydroturbine is prone to abrasion during service, and the change of residual stress at this position has a direct impact on the failure of the blade. Therefore, there is a great need to investigate the residual stress variation at this location. Figure 16 indicates the variation of the instantaneous residual stresses at the characteristic point near the lower ring of the blade water outlet in both welding sequences with the welding time when the preheating temperature is 150 °C. It can be found that the variation law of residual stress at feature points under the two welding sequences is basically the same. Path B starts arcing near this position, so the instantaneous stress change gradient is very large at the beginning of welding. When the heat source passes through this location, the metal in the feature point and its vicinity melts, and the residual stress is reduced instead. After the heat source passes through this position, the area begins to cool with time, and the residual stress increases rapidly. When cooling to a certain time, the change of residual stress gradually becomes smooth. When the workpiece is completely cooled, the residual stress at the characteristic point of the water outlet edge of Path A blade is about 731.2 MPa. However, the residual stress at the characteristic point of the water outlet edge of the Path B blade is about 649.1 MPa. This also shows that the welding sequence of the three-stage welding (Path B) can reduce the residual stress.

## 6. Conclusions

In summary, the abraded area of the hydraulic turbine blade and the lower ring connection were repaired by overlay welding. Numerical simulation and experimental verification were used to investigate the influence of the welding sequence and preheating temperature on the temperature and residual stress distribution of the overlay joint. The temperature distribution and residual stresses at different locations were compared and analyzed. The main findings of the results of this study are as follows:

1. Through checking the welding finite element model, it was found that the transient thermal cycling and welding residual stress experimental values and numerical simulation values match well, and the distribution trend is consistent, proving the effectiveness of the welding finite element model developed in this paper.
2. Under the influence of welding repair, the overall distribution of welding stress is not uniform, the high-stress area is predominantly focused on the weld and adjacent areas, and the stress gradient is large.
3. The welding sequence is one of the important factors impacting the distribution trend of welding residual stresses. Under the welding sequence of continuous welding (Path A), the residual stress is 731.2 MPa at the water outlet side of the blade near the lower ring. Under the welding sequence of three-stage welding (Path B), the residual stress is 649.1 MPa at the water outlet side of the blade near the lower ring. The difference between the two is 82.1 MPa. Therefore, the sequence of the weld overlay effect is as follows: the three-stage welding > the continuous welding.
4. The welding preheating temperature is the major factor impacting the maximum value of residual stress in welding. The peak value of weld residual stress in the three-stage welding sequence (Path B) gradually decreased from 829.58 MPa to 796.29 MPa while the preheating temperature was raised from 50 °C to 150 °C. Therefore, a reasonable preheating temperature can effectively reduce welding residual stress.
5. The longitudinal residual stress is bigger than the transverse residual stress, and the residual stress on the water outlet side of the blade is higher than the water inlet surface. The transverse residual stress of the blade water outlet surface is tensile stress. However, the direction of the transverse residual stress of the blade water inlet surface is completely opposite to the direction of the transverse residual stress of the blade water outlet surface, but the distribution trends are similar.

## Figures and Tables

**Table 1:** Compositions of 0Cr13Ni5Mo steel and 0Cr13Ni5MoRe (mass fraction, %).

Materials	C	Si	Mn	P	S	Cr	Ni	Mo
0Cr13Ni5Mo	0.02	0.46	0.61	0.035	0.007	13.4	5.03	0.77
0Cr13Ni5MoRe	0.014	0.36	0.78	0.022	0.01	12.5	4.33	0.0585

**Table 2:** Welding Cases.

Case	Welding Sequence	Preheating Temperature/°C
Case 1	Path A	50
Case 2	Path A	100
Case 3	Path A	150
Case 4	Path B	50
Case 5	Path B	100
Case 6	Path B	150

## Data Availability

Not applicable.
